# A new species of *Pseudaulacaspis* MacGillivray, 1921 from China (Hemiptera, Coccoidea, Diaspididae) with a key to Chinese species

**DOI:** 10.3897/zookeys.210.3122

**Published:** 2012-07-24

**Authors:** Jiu-Feng Wei, Ji-Nian Feng

**Affiliations:** 1 College of Life Sciences, Northwest A & F University; 2 Key Laboratory of Plant Protection Resources and Pest Management, Ministry of Education, Entomological Museum, Northwest A & F University, Yangling, Shaanxi Province, 712100, China

**Keywords:** Hemiptera, armored scale, taxonomy, Diaspididae, new species

## Abstract

A new species of armored scale, *Pseudaulacaspis zhenyuanensis* Wei & Feng, **sp. n.** is described and illustrated from specimens collected on *Spermadictyon suaveolens* in China. A key to armored scale species known from China is provided.

## Introduction

The Coccoidea is one of the four superfamilies of the monophyletic suborder Seternorrhyncha belongs to the Hemiptera (Gullan and Cook 2007), with at least 30 families and around 8000 species (Andersen 2010). The family Diaspididae is the largest family of the Coccoidea with more than 2400 dispidid species currently known (Ben-dov 2012). The higher classification within the family is uncertain but two of the major subfamilies are the Aspidiotinae and the Diaspidinae, and most species can be assigned to one or the other (Miller and Davidson 2005)

The genus *Pseudaulacaspis* was established by MacGillivray (1921) for *Diaspis pentagona* Targioni Tozzetti, 1886 was belongs to subfamily Diaspidinae. When he described it, he referred to it 9 nominal species, which are now considered to represent only 2 species. Since then, many additional species were described and added to *Pseudaulacaspis* by other authors (Chen 1983; Ferris 1953, 1955; Hu 1986; Takagi 1956, 1961, 1966, 1970, 1985; Tang 1986, 1988; Williams and Watson 1988; Hodgson and Lagowska 2011). This genus is large with 68 species (Hodgson and Lagowska 2011) which is a widespread and polyphaous genus infesting a large number of plant (Borchsenius 1966) and occurs in most of zoogeographical regions except Antarctica. Up until now, 32 species have been described from China.

In the present paper, a new species *Pseudaulacaspis zhenyuanensis* sp. n.is described and illustrated, bringing the number of recognized species in this genus to 69, of which 33 are recorded from China. And a key to species from China is included.

## Materials and methods

The morphological terms for Diaspididae follow those of Henderson (2011). The illustrations of the adult female are drawn from slide-mounted specimens, which depict the dorsum on the left and venter on the right. Enlargements of important characters are shown around the edges of the main illustration. All measurements are given in micrometers (μm). Measurements were made using the measurement tools NIT-Elements D. The abbreviations L1, L2, L3 and L4 stand for median and second to fourth pygidial lobes.

All specimens are deposited in the Entomological Museum, Northwest A & F University, Yangling, Shaanxi, China (NWAFU).

## Taxonomy

### Genus Pseudaulacaspis

MacGillivray, 1921

http://species-id.net/wiki/Pseudaulacaspis

Pseudaulacaspis MacGillivray, 1921: 305. Type species: *Diaspis pentagona* Targioni Tozzetti, by original designation.

#### Generic diagnosis.

**Female scale.** White, suborbicular or long pyriform. Exuviae terminal. **Male scale.** Same colour as female scale, elongate.

**Adult female.** Body shape varied, fusiform, olivary or elongate; derm membraneous except for the marginal of pygidium; mesothorax, metathorax, and abdominal segments I-III produced laterally. **Cephalothorax.** Antennae each with a seta. Anterior spiracles each usually with a cluster of trilocular pores, posterior spiracle each associate with or without trilocular pores. **Pygidium.** With 2 or 3 pairs of lobes. Median lobes (L1) well-developed, much larger than lobules of lateral lobes, zygotic basally, with a distinct pair of marginal setae between lobes. In general, L1 divide into two types: bark-type, individuals occur on bark and prominent median lobes; leaf-type, those on leaves and sunken into the pygidium. Second lobes (L2) much smaller than the L1, bilobed, divided into inner lobule and outer lobule, outer lobule usually smaller than inner, in some species much reduced. Third lobes (L3) smaller than L2, bilobed or represented by serrations along the body margin in some species. **Gland spines.** Gland spines developed, usually single on abdominal segments VI-VIII, becoming shorter into conical on anterior segments which called gland tubercles. **Ducts.** Dorsum with 2-barred ducts, forming submedial and submarginal rows on abdominal and pygidium, usually as same size as marginal macroducts. Ventral microductsscattered. **Anal opening.** Anal opening close to the base of or situated about the centre of the pygidium. **Perivulvar pores** quinquelocular, in five groups.

#### Remarks.

This genus is very closely related to *Chionaspis* Signoret, 1868 and *Aulacaspis* Cockerell, 1893 in feature of pygidial lobes and dorsal ducts present on pygidium and abdomen, but can differ from these genus: presence of a pair of setae between the median lobes in *Pseudaulacaspis*, but absent in *Chionaspis* and *Aulacaspis*.

### Pseudaulacaspis zhenyuanensis

Wei & Feng sp. n.

urn:lsid:zoobank.org:act:AB75F5D8-7BDF-42D4-9DA9-13F4DFD1D43F

http://species-id.net/wiki/Pseudaulacaspis_zhenyuanensis

Figures 1–8

#### Material examined.

**Holotype:** adult female:CHINA:Guizhou Prov., Zhenyuan County, 13. viii. 1996, Zeng (NWAFU).

Paratypes: 2 adult females: same data as the holotype (NWAFU).

#### Description

**, n=3. Adult female.** Appearance in life not recorded. Slide-mounted adult female 1755-1910 μm long (holotype 1910 μm long); 930-970 μm wide (holotype 931 μm wide), body outline fusiform, derm membranous except for pygidium. Normally widest at metathorax and abdominal segment I, lateral abdominal lobes well-developed, with large gland spines on the margin of prepygidial and pygidial segments. **Cephalothorax.** Antennae each with 1 long fleshy seta, distance between antennae is 111 µm**.** Anterior spiracle each with 12-31 trilocular pores in a cluster, posterior spiracle each with 11-17 trilocular pores. **Pygidial Lobes.** With 3 pairs of lobes; L1 well-developed, zygotic basally, protruding from pygidial margin, with small serrations along both margins, with a pair of setae between lobes; L2 bilobate, inner lobule rounded, much larger than outer lobule; L3 bilobate, slightly smaller than L2, inner lobule rounded, outer lobule margin serrate; L4 represented by serrations along the body margin. **Gland spines.** Large, arranged singly on pygidial segments VI-VIII but with 2 on segment V, 4-5 on segment IV, 5-6 on segment III, 5 on segment II, anterior spines smallest (on segment II). **Gland tubercle** present submarginally, with 2 on prothorax, 9-11 on mesothorax, 5-6 on metathorax, 6 on segment I. **Ducts.** Marginal macroducts, 2-barrel-shaped, 1 present between L1 and L2, 2 on segment VI, 1 on segment V. Dorsal macroducts on pygidium about same size as marginal macroducts, becoming slightly smaller on anterior abdomen, 2-barrel-shaped, arranged segmentally in submedian and submarginal rows; submedian: 3-6 on segment I, 4-5 on II, 4-6 on III, 7-13 on IV, 3-4 on V; submarginal: 11-14 on I, 11-12 on II, 10-11 on III, 10-11 on IV, 8-9 on V. Dorsal ducts scattered on margin of thorax, smaller than those on abdomen, 2-barrel-shaped, with 8 or 9 on prothorax, 15-17 on mesothorax, 14 or 15 on metathorax. Dorsal ducts on head as big as ventral microducts, very smaller than dorsal ducts present on thorax, scattered distribution. Ventral microductsscattered, numerous on head and with several microducts on submargin of pygidium and prothorax and submedian of abdomen, meso- and metathorax. **Anal opening,** small, 15-17µm in diameter, positioned 214 µm from posterior margin. **Perivulvar pores** in 5 groups, 31-37 in the median group, 33-44 in the anterolaterally and 45-48 in the posterolaterally.

#### Diagnosis.

This species is similar to *Pseudaulacaspis chinensis* (Cockerell, 1896) in body shape and the number of pygidial lobes, but can be distinguished by the following features (those for *Pseudaulacaspis chinensis* in brackets): 1) dorsal macroducts absent on abdominal segment VI (present); 2) L1 prominent the pygidium (sunken into the pygidium).

Host: *Spermadictyon suaveolens*.

#### Etymology.

The specific epithet is named after Zhenyuan, the type locality.

#### Distribution.

China (Guizhou).

**Figures 1–8.**
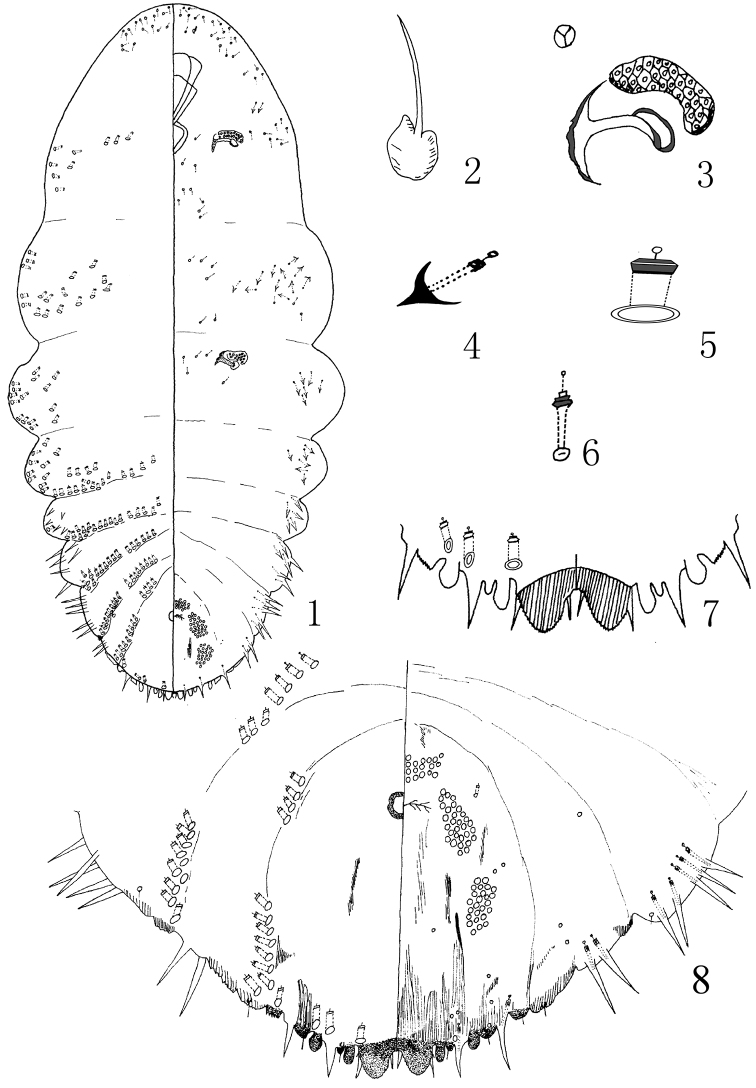
Pseudaulacaspis zhenyuanensis Wei & Feng, sp. n., adult female: **1** habitus **2** antennae **3** anterior spiracle **4** gland tubercles **5** detail of gland macroduct **6** detail of the duct in the head on the dorsum **7** detail of pygidium **8** pygidium.

#### Key to Chinese species of the genus *Pseudaulacaspis*

**Table:** 

1	Trilocular pores absent near each anterior spiracle	*Pseudaulacaspis manni* (Green & Mann, 1907)
–	Trilocular pores present near each anterior spiracle	2
2	Body slender, both side nearly parallel	*Pseudaulacaspis dendrobii* Kuwana & Muramatsu, 1931
–	Body nonslender	3
3	Body suborbicular or oval	4
–	Body long ovate or furiform	8
4	Trilocular pores present near each anterior spiracle, absent near each posterior spiracle	5
–	Trilocular pores present near anterior spiracle and posterior spiracle	7
5	With 2 pairs of lobes on pygidium	*Pseudaulacaspis canarium* Hu, 1986
–	With 3 pairs of lobes on pygidium	6
6	The eggs white or salmon; with 1 pairs of gland spines between L3 and the traces of L4, each bifurcate	*Pseudaulacaspis pentagona* (Targioni Tozzetti, 1886)
–	The eggs always salmon; with 2 pairs of gland spines between L3 and the traces of L4, each pointed	*Pseudaulacaspis prunicola* (Maskell, 1895)
7	Perivular pores in 6 groups	*Pseudaulacaspis mirabilis* Hu, 1986
–	Perivular pores in 5 groups	*Pseudaulacaspis ficicola* Tang, 1986
8	Anterior spiracle and posterior spiracle both with trilocular pores	9
–	Anterior spiracle with trilocular pores, posterior spiracle without trilocular pores	14
9	Dorsal macroducts absent on submarginal and submedial area of abdominal segment VI	10
–	Dorsal macroducts present on submarginal or submedial area of abdominal segment VI	11
10	Submarginal and submedial macroducts present on abdominal segment I	*Pseudaulacaspis zhenyuanensis* sp. n.
–	Submarginal and submedial macroducts absent on abdominal segment I	*Pseudaulacaspis ulmicola* Tang & Li, 1988
11	Dorsal macroducts absent on submarginal and submedial area of abdominal segment II	12
–	Dorsal macroducts present on submarginal or submedial area of abdominal segment II	13
12	Antennae 6 segments in first instar	*Pseudaulacaspis centreesa* (Ferris, 1953)
–	Antennae 5 segments in first instar	*Pseudaulacaspis eucalypticola* Tang, 1986
13	Dorsal macroducts present on submedial area of abdominal segment VI; anal opening situated at the base of pygidium	*Pseudaulacaspis momi* (Kuwana, 1931)
–	Dorsal macroducts present between submarginal or submediaal area of abdominal segment VI; anal opening situated on the centre of pygidium	*Pseudaulacaspis loncerae* Tang, 1986
14	Dorsal macroducts present on submarginal or submedial area of abdominal segment VI	15
–	Dorsal macroducts absent on submarginal and submedial area of abdominal segment VI	23
15	Dorsal macroducts present on submarginal and submedial area of abdominal segment VI	16
–	Dorsal macroducts only present on submedial area of abdominal segment VI	18
16	L2 bilobate, each with a pair of short basal scleroses	*Pseudaulacaspis sasakawai* Takagi, 1970
–	L2 bilobate, without basal scleroses	17
17	With 9-11 trilocular pores near each anterior spiracle	*Pseudaulacaspis camelliae* (Chen, 1983)
–	With more than 25 trilocular pores near each anterior spiracle	*Pseudaulacaspis latisoma* (Chen, 1983)
18	Dorsal macroducts present on submarginal or submedial area of abdominal segment I	19
–	Dorsal macroducts absent on submarginal or submedial area of abdominal segment I	20
19	L3 not obvious, anal opening situated on the centre of pygidium	*Pseudaulacaspis takahashii* (Ferris, 1955)
–	L3 bilobate, anal opening situated at the base of 2/5 of pygidium	*Pseudaulacaspis chinensis* (Cockerell, 1896)
20	L3 bilobate	21
–	L3 not obvious, present by a prominence	22
21	L1 protruding from pygidial margin	*Pseudaulacaspis cockerelli* (Cooley, 1897)
–	L1 sunk into apex of pygidium	*Pseudaulacaspis kentiae* (Kuwana, 1931)
22	Only 1 submedial macroduct present on abdominal segment VI	*Pseudaulacaspis eugeniae* (Maskell, 1892)
–	With 2 submedial macroducts present on abdominal segment VI	*Pseudaulacaspis ericacea* (Ferris, 1953)
23	Submarginal macroducts present on abdominal segment II, submedial macroducts absent on abdominal segment II	24
–	Submarginal and submedial macroducts both present on abdominal segment II	26
24	Submedial macroducts absent on abdominal segment III	*Pseudaulacaspis subcorticalis* (Green, 1905)
–	Submedial macroducts present on abdominal segment III	25
25	With more than 30 trilocular pores near each anterior spiracle	*Pseudaulacaspis poloosta* (Ferris, 1953)
–	With 20 or fewer trilocular pores near each anterior spiracle	*Pseudaulacaspis megaloba* (Green, 1899)
26	L3 obvious, bilobate	27
–	L3 not obvious, present by a shallow prominence	28
27	With 2-4 trilocular pores near each anterior spiracle	*Pseudaulacaspis subrhombica* (Chen, 1983)
–	With 11-14 trilocular pores near each anterior spiracle	*Pseudaulacaspis frutescens* (Hu, 1986)
28	With 10 or fewer trilocular pores near each anterior spiracle	29
–	With more than 15 trilocular pores near each anterior spiracle	31
29	L1 protruding from pygidial margin, only with 2 trilocular pores near each anterior spiracle	*Pseudaulacaspis taiwana* (Takahashi, 1935)
–	L1 sunk into apex of pygidium, with more than 4 trilocular pores near each anterior spiracle	30
30	The terminal of L3 arc-shaped, smoothly, with 4-8 trilocular pores near each anterior spiracle	*Pseudaulacaspis abbrideliae* (Chen, 1983)
–	The terminal of L3 serration, with more than 4 trilocular pores near each anterior spiracle	*Pseudaulacaspis brideliae* (Takahashi, 1933)
31	Gland tubercles present on prothorax, dorsal ducts present on head, smaller than those present on pygidium and abdomen	*Pseudaulacaspis syzygicola* (Tang, 1986)
–	Gland tubercles absent on prothorax, dorsal ducts absent on head	32
32	L2 small, bilobulate, with 17-20 submedial macroduct in total	*Pseudaulacaspis kuishiuensis* (Kuwana, 1909)
–	L2 very small, the outer lobule at times almost obsolete, with 4-11 submedial macroduct in total	*Pseudaulacaspis celtis* (Kuwana, 1928)

## Supplementary Material

XML Treatment for Pseudaulacaspis

XML Treatment for Pseudaulacaspis zhenyuanensis
